# Improving Human Papilloma Virus Vaccination Rates at an Urban Pediatric Primary Care Center

**DOI:** 10.1097/pq9.0000000000000098

**Published:** 2018-09-19

**Authors:** Nicola Brodie, Katie E. McPeak

**Affiliations:** From the Department of Pediatrics, St. Christopher’s Hospital for Children, Drexel University College of Medicine, Philadelphia, Pa.

## Abstract

**Introduction:**

Despite compelling evidence regarding its safety and efficacy, human papilloma virus vaccination rates remain low nationally with high rates of missed vaccination opportunities. Provider recommendation is the most important factor in determining vaccine approval by families; yet, studies show that providers are hesitant to strongly recommend vaccination, especially at younger ages. We hypothesized that educational and quality improvement interventions targeting our clinical team would decrease rates of missed opportunities to vaccinate patients aged 11–13 years and improve vaccination rates among patients aged 9–10 years old.

**Methods:**

This quality improvement project took place at an urban, academic pediatric primary care center in north Philadelphia, which serves as the medical home for over 22,000 patients. A multidisciplinary team performed a series of planned sequential interventions to improve human papilloma virus vaccination rates. The electronic health records of children aged 9–13 who presented to our center from September 2014 through December 2015 were queried. Statistical process control charts and established rules for detecting special cause variation were applied.

**Results:**

Rates of missed opportunities to vaccinate 11- to 13-year-old patients decreased from 63% to 18% during the intervention period. Rates of immunization of 9- to 10-year-old patients increased from 56% to 84% during the intervention period.

**Conclusion:**

This low-cost, multifaceted, interdisciplinary quality improvement project resulted in a decrease in missed opportunities to vaccinate among children aged 11–13 years old and improved the vaccination rates of 9–10 year olds. Ongoing interventions are needed to sustain these efforts and to ensure timely vaccine series completion.

## INTRODUCTION

Human papilloma virus (HPV) is the most common causative agent of cervical cancer, and cancers of the oropharynx, anus, and vulva. HPV is the most prevalent sexually transmitted infection, with about 14 million new infections per year in the United States.^1^ The highest risk of HPV infection is shortly after sexual debut with 75% of new infections occurring in 15–24 year olds.^1^ Cervical cancer disproportionately affects women of low socioeconomic status, with high morbidity and mortality noted in this population.

Given the risks of cervical and other cancers, the HPV vaccine was developed as a tool for cancer prevention. The HPV vaccine was approved for use in females in June 2006, and the quadrivalent vaccine was approved for use in males in 2009. In 2014, a nine-valent vaccine was approved for use in both males and females. The vaccine is approved for use in people aged 9–26 years (with recommendations for initiation at age 11), in a 2 or 3 dose series, depending on age of vaccine initiation. At the time of this study, a 3 dose immunization series was recommended for all patients, regardless of age. Since study completion, these recommendations have changed to reflect a new 2 dose series for younger patients.^2^

Due to persistently low rates of vaccination,^3^ many studies have been conducted to assess barriers to HPV vaccine uptake, both from the perspective of parents and from the perspective of vaccine providers. Much research has been done regarding parental and provider attitudes toward vaccination, and the barriers faced by parents and providers in achieving target rates of vaccine series completion.^4–15^ These barriers include fewer medical home visits in patients of the target age group, the vaccine not being required for school attendance, parental and provider concerns regarding sexuality and HPV vaccination, and a lack of knowledge about the vaccine by both parents and providers.^8,11,12^

The importance of a strong provider recommendation to vaccinate has been well documented in the literature.^4,10,12,14,15^ The Centers for Disease Control provides guidelines for providers on a strong recommendation that includes recommending the HPV vaccine at every eligible clinical encounter, recommending in the same way as other adolescent vaccines, emphasizing the role of the vaccine in cancer prevention, and highlighting a personal belief in vaccine effectiveness and safety.^16^ Using these criteria to define a quality recommendation, 1 recent study found that parents who reported receiving a high-quality recommendation from their provider were 9 times more likely to initiate vaccination.^17^

In addition to the importance of a strong provider recommendation, missed vaccination opportunities also play an important role. In 1 study of 16 urban, academically affiliated ambulatory pediatric practices, nearly 3 quarters of unvaccinated subjects had experienced at least 1 missed vaccination opportunity and mean age at vaccine initiation was about 16 years.^5^ Younger adolescents (11–12 year olds) were more likely to have missed opportunities. Another study conducted by the Centers for Disease Control using National Immunization Survey-Teen data found that if all missed opportunities for HPV vaccination had been eliminated, over 90% of girls would have received at least 1 dose of the vaccine by age 13.^3^

With a foundation in the literature that suggested that a strong provider recommendation to vaccinate is a key determinant in vaccine uptake, and that patients who are vaccinated at younger ages and are offered vaccination at every clinical encounter (including acute care visits) are more likely to achieve series completion before sexual debut, we undertook a large-scale and interdisciplinary quality improvement project to increase our rates of HPV vaccine administration and decrease our rates of missed opportunities to vaccinate.

Our global aim was to improve our HPV vaccination rates by educating our clinical staff to provide a strong recommendation to vaccinate; vaccinate at every clinical encounter at our practice; and initiate vaccination starting at age 9. Our specific aims were 2-fold:

1) To decrease the rate of missed opportunities to vaccinate 11–13 year olds presenting to the Primary Care Center for the Urban Child at St. Christopher’s Hospital for Children (CUC) to 20% by April 1, 2015
2) To increase the rate of HPV vaccine initiation among 9–10 year olds presenting to the CUC to 80% by September 1, 2015

## METHODS

### Setting

Our primary care practice is located in a socio-economically disadvantaged urban neighborhood in North Philadelphia. The CUC cares for over 22,000 patients up to the age of 22. Our payer mix consists of greater than 85% Medicaid patients; hence, vaccination is predominantly achieved through the Vaccines for Children program. Our patients are at particularly high risk for early HPV infection as data suggests that 10% of Philadelphia teens have had sexual intercourse before age 13 (about 2.5 times the national average).^18^ Additionally, our patients are at high risk for the sequelae of HPV infection, particularly cervical cancer, as the rate of cervical cancer in Philadelphia exceeds the national rate by greater than 40%.^19^

### Baseline Data

Preliminary data available before our intervention period showed that CUC vaccination practices were aligned with trends noted in the literature. Based on Vaccines for Children data, before our intervention, HPV vaccine initiation rate was approximately 79%, with a series completion rate of only 32% by age 18. Approximately 40% of patients experienced missed opportunities for HPV vaccination, and 76% of patients were not vaccinated within the recommended time interval. Only 8% of our patients received 3 doses of the vaccine by age 13.^20^

### Design

This study was designed as a quality improvement project with multiple sequential interventions. Analysis was conducted using statistical process control charts, and established rules for detecting special cause variation were used.^21^ Data were obtained monthly by a query of our electronic health record.

During the first plan-do-study act (PDSA) cycle, a survey was administered to all members of our clinical team (inclusive of key nonclinical front line staff, medical assistants, nurses, nurse practitioners, residents, and attending physicians) to assess baseline HPV vaccine knowledge and perceived barriers to vaccination. This survey was a validated 22-item questionnaire adapted with permission from the HPV HINTS Survey.^15^ The survey assessed HPV disease knowledge, HPV vaccine knowledge, and perceived barriers to vaccination. Barriers to vaccination were assessed using 10 questions with Likert-scale formatting with total scores ranging from 10 to 50. Barriers among members of the clinical team were compared using a Wilcoxon rank sum test to adjust for the skewed distribution of survey responses.

The results of this survey were used to assemble the intervention team. The entire clinical team was educated regarding the HPV vaccine and how to provide a strong recommendation to vaccinate at every clinical encounter. Two 30-minute didactic sessions were offered to all members of the clinical staff, including pediatric residents, attending physicians, nurse practitioners, medical assistants, and office practice service coordinators. The intervention team included a data scientist, a resident physician, an attending physician clinical champion, and the medical director—an attending physician with significant quality improvement expertise, and perhaps most importantly a medical assistant champion who was instrumental in shifting the culture of our institution.

**Fig. 1.**
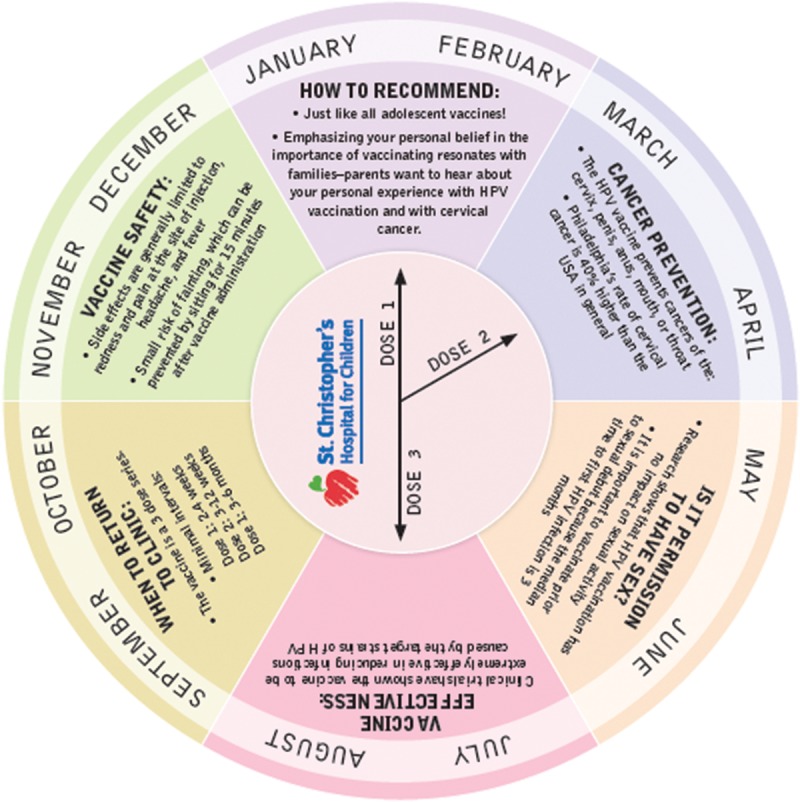
HPV vaccine wheel tool. During the second PDSA cycle, a practical interactive wheel tool was distributed to providers to assist in vaccine recommendation and appropriate recall for the next vaccine dose.

In the second PDSA cycle, weekly reminders were disseminated via e-mail to the clinical team with important facts about HPV and the importance of HPV vaccination; these reminders were reinforced during preexisting team briefs before each clinic session. Additionally, during this phase, a wheel tool was developed and distributed with validated information designed to help providers appropriately recommend the vaccine. The moving inner wheel allowed providers and office staff to easily calculate the timing of subsequent vaccine doses to ensure the timely recall of patients to receive the next dose (Fig. 1).

To assess outcomes, the rates of missed opportunities to vaccinate patients aged 11–13 were tracked monthly. A missed opportunity was defined as an eligible patient who did not receive the HPV vaccine at the encounter in question. The rates of vaccine initiation in patients aged 9–10 were also tracked each month through the duration of the study period (September 2014 to December 2015). Patients were included in this analysis if they were aged 9–10 and presented to our clinic for any reason. Patients who received the HPV vaccine comprise the numerator of our analysis. These data were tracked by a monthly query of our electronic health record, where all visits of patients in the relevant age range were assessed for the presence or absence of the Current Procedural Terminology code for HPV vaccination.

This study was approved by the Drexel University Institutional Review Board.

## RESULTS

**Fig. 2.**
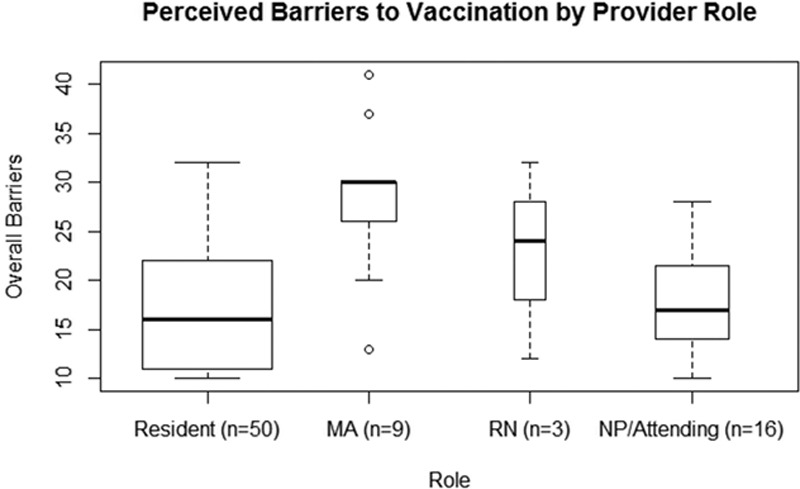
Perceived barriers to vaccination. Barriers to vaccination were assessed by 10 questions with Likert scale formatting. A high total barrier score represents more perceived barriers to vaccination. Scores ranged from 10 to 50. Results were analyzed using a Wilcoxon rank sum test to adjust for the skewed distribution of the data. Compared with residents, medical assistants perceived more barriers to vaccination (*P* = 0.012). There was no significant difference between residents and attending physicians with regard to barriers (*P* = 0.52).

A total of 86 (65%) of 132 members of the clinical team completed the HPV HINTS survey. Survey scores for barriers to HPV vaccination ranged from 10 to 50. We found that medical assistants perceived more barriers to vaccination in comparison to residents and attending physicians, with a median barrier score of 30 compared with a barrier score of 15 (*P* = 0.012; Fig. 2).

**Fig. 3.**
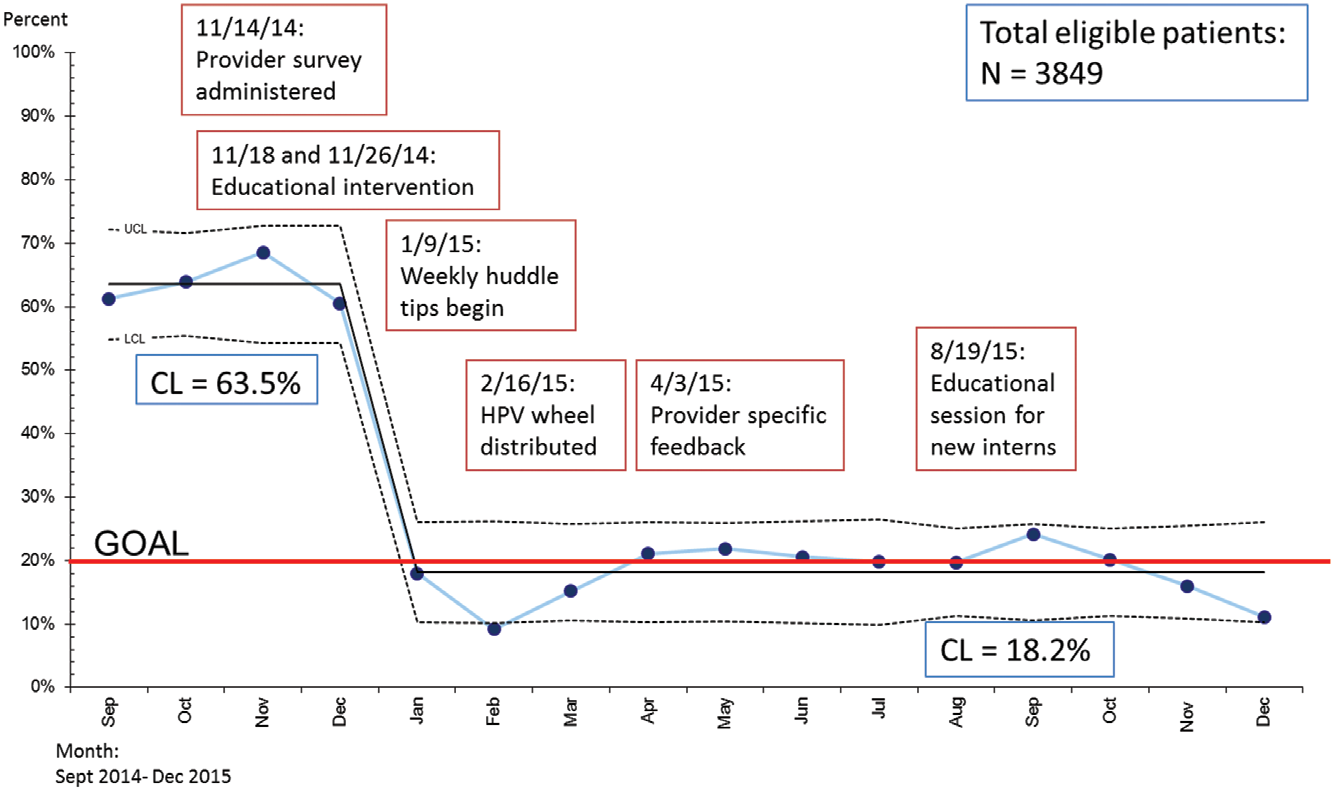
Rates of missed opportunities to vaccine. Rates of missed opportunities to vaccinate patients who were not fully immunized ages 11–13 presenting for acute or well-child care at the CUC were plotted on a statistical process control chart with an upper control limit and lower control limit calculated and depicted above. This rate is plotted in relation to interventions conducted during the study period. Established rules for detecting special cause variation were applied and the CL was adjusted from 65.5% to 18.2% during the study period. CL, center line.

Missed opportunities to vaccinate and rates of vaccination of patients were analyzed using statistical process control charts. There were 3,849 patient encounters for patients aged 11–13 who had received fewer than 3 doses of the HPV vaccine occurred during the study period. Patients were included in this analysis if they were aged 11–13 and presented to our clinic for any reason, including sick care, and had not yet completed the HPV vaccine series. Missed opportunities to vaccinate decreased from 65% in the preintervention period to 18% during the intervention period (Fig. 3). A special cause was detected immediately following the implementation of the educational intervention and start of our huddles.^22^

**Fig. 4.**
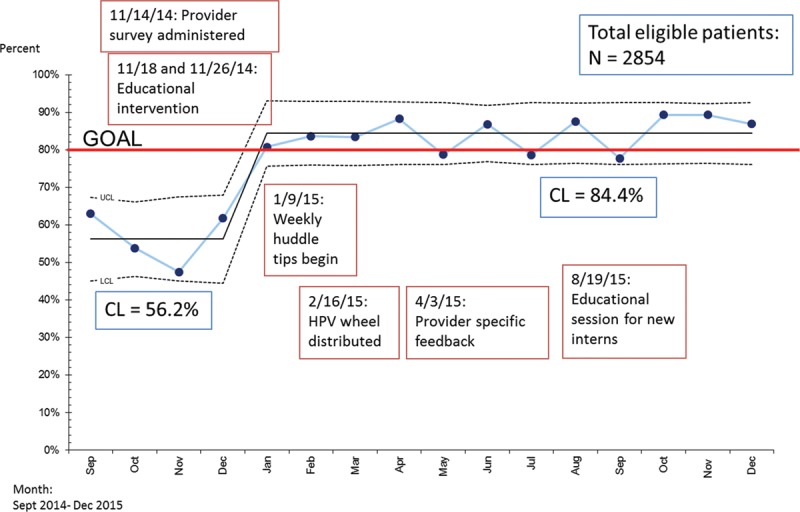
Rates of vaccine initiation. Rates of vaccine initiation for patients aged 9–10 presenting for acute or well-child care at the CUC were plotted on a statistical process control chart with an upper control limit and lower control limit calculated and depicted above. This rate is plotted in relation to interventions conducted during the study period. Established rules for detecting special cause variation were applied and the CL was adjusted from 56.2% to 84.4% during the study period. CL, center line.

There were 2,854 patient encounters for patients aged 9–10 occurred during the study period. A significant increase in vaccine initiation rates (56–84%) occurred in patients aged 9–10 years of age (Fig. 4).^22^ Statistical process control charts are depicted in Figs. 3, 4 with a depiction of the monthly rates of immunization in relation to our sequential interventions (Figs. 3, 4).

## DISCUSSION

This quality improvement project yielded important insights and resulted in significant improvements in HPV vaccination rates in our practice. We noted that medical assistants reported significantly more barriers to vaccinating our patients than physician and Certified Registered Nurse Practitioner (CRNP) providers. Although this finding did not change the content of our education intervention, it highlighted the importance of including medical assistants in our intervention and incorporating their voice in the improvement team. The role of medical assistant recommendation or hesitation has not been explored in the literature to date. Further studies are needed to obtain a more detailed understanding of the barriers perceived and determine their influence on patient outcomes.

During the intervention stages of our project, we were able to decrease our rate of missed opportunities to vaccinate in patients aged 11–13 from 64% to 18%. This population was specifically targeted, given the need to ensure complete immunity before age 13 in our vulnerable patient population. The standard of care in our practice is now to offer vaccination at every eligible clinical encounter, including acute care visits. To ensure that our success is sustainable, yearly educational interventions take place with new resident physicians and new providers are educated during their orientation period. It is likely that this culture shift has likely increased the rates of timely immunization for other vaccines; however, additional research is necessary to assess this potential positive impact of our study.

Our findings are in keeping with a recent quality improvement study in a similar population in urban Denver. This study found that a similarly low-cost intervention designed to normalize the HPV vaccine and recommend the vaccine at every clinical encounter led to statistically significant increases in both vaccine administration and in series completion.^23^ These findings suggest that initiatives undertaken in our practice are not limited to our geographical region and are likely generalizable at least to other practices comprising patients of a similar socioeconomic profile.

Finally, we were able to increase our rates of vaccination of our patients aged 9–10 from 56% to 84%. This finding suggests that parents are amenable to early vaccination so long as a provider recommends the vaccine. Ours is the first study in the United States to our knowledge to study the impact of vaccine recommendation at extremely young ages. Although waning immunity over time is a potential risk factor in very early vaccination, studies to date support an improved immunogenic response from younger pre-teens and teens compared with young adults.^24–26^ Given the predilection for early sexual debut in our population, vaccinating as early as possible will allow for full immunity in a timely fashion. Many providers reported that vaccinating at earlier ages was easier than older ages, as sexual activity is still a distant idea in the minds of patients and their care-takers, and thus the conversation can focus on cancer prevention rather than the sexual transmission of HPV. This finding is still anecdotal and merits further study. Additionally, ongoing investigation is needed to assess whether earlier vaccine administration does lead to vaccine series completion at a younger age; an anecdotal review of patient charts suggests that it does.

This study has several limitations, including limitations to generalizability and confounding initiatives, which may have influenced results. Our hospital has a strong culture of quality improvement and perhaps our interventions would not be successful in a different clinical setting. This is particularly true as most of our interventions were low-reliability or highly user dependent. Additionally, our population is at particularly high risk for HPV infection and for cervical cancer later in life. Patients and families in lower risk populations may be more hesitant to initiate vaccination so early. Finally, during our study period, the Philadelphia Department of Public Health embarked on a media campaign to increase HPV vaccine initiation and series completion, which likely contributed to our results.

## CONCLUDING SUMMARY

Quality improvement methodology can be used to successfully increase the rate of HPV vaccination in an urban pediatric primary care practice. Implementation of routine vaccination at acute care visits and vaccine administration at younger ages were key to the improvement in vaccination rates and to decreasing rates of missed opportunities to vaccinate. Future studies should be conducted to assess the generalizability of early vaccine administration. Additional studies are needed to assess the impact of the new recommended vaccine administration schedule on timely vaccine series completion.

## ACKNOWLEDGMENTS

Assistance with the study: the authors acknowledge Nacoyia Bey for her substantial contribution to the study.

## DISCLOSURE

The authors have no financial interest to declare in relation to the content of this article.
